# Definitive chemoradiotherapy plus immune checkpoint inhibitors for locally advanced unresectable esophageal squamous cell carcinoma: survival and progression patterns in a propensity‐matched cohort

**DOI:** 10.3389/fimmu.2026.1778539

**Published:** 2026-05-28

**Authors:** Zhongmei Lin, Yongshi Shen, Qin Li, Daojia Liu, Juhui Chen, Siqian Cai, Zhizhong Lin, Mingqiu Chen, Yu Lin, Junqiang Chen, Yuanji Xu

**Affiliations:** 1 Department of Radiation Oncology, Clinical Oncology School of Fujian Medical University, Fujian Cancer Hospital, Fuzhou, Fujian, China; 2 Department of Intensive Care Unit, Clinical Oncology School of Fujian Medical University, Fujian Cancer Hospital, Fuzhou, Fujian, China; 3 Department of Nuclear Medicine, Clinical Oncology School of Fujian Medical University, Fujian Cancer Hospital, Fuzhou, Fujian, China

**Keywords:** chemotherapy, esophageal squamous cell carcinoma, immunotherapy, prognostic factors, progression, survival

## Abstract

**Background:**

Locally advanced unresectable esophageal squamous cell carcinoma (ESCC) remains a therapeutic challenge, with limited survival outcomes following definitive chemoradiotherapy (CRT). Recent evidence suggests that combining immune checkpoint inhibitors (ICIs) with CRT may enhance therapeutic response. Nevertheless, its efficacy, progression behavior, and prognostic indicators require further elucidation.

**Methods:**

A retrospective review was performed on ESCC patients treated at Fujian Cancer Hospital from August 2019 to August 2024. 67 patients receiving definitive CRT combined with ICIs (CRT+ICIs) were compared with 415 historical controls treated with CRT alone. To correct for baseline imbalances, 1:3 propensity score matching (PSM) was applied. The Kaplan-Meier method was employed to assess survival outcomes, while progression patterns were evaluated via Fine-Gray competing risks model. Cox proportional hazards analyses were used to determine prognostic factors for overall survival (OS).

**Results:**

After PSM, 62 individuals were retained in the CRT+ICIs group and the matched CRT group included 149. The CRT+ICIs exhibited a significantly prolonged median OS (31.1 vs. 18.6 months, p = 0.013) and showed a tendency toward longer PFS (18.6 vs. 12.1 months, p = 0.176). Fine-Gray analysis indicated a nonsignificant 34% reduction in distant progression for CRT+ICIs group (p = 0.160), while local-regional progression risk was declined over time. In the CRT+ICIs group, multivariable analysis identified M stage, lactate dehydrogenase-to-albumin ratio (LAR) and prognostic nutritional index (PNI) as independent prognostic factors for OS. Among these patients, 38.7% experienced grade 3 or higher adverse events with manageable immune-related toxicity.

**Conclusions:**

CRT combined with immunotherapy significantly prolongs OS and may delay early distant progression in locally advanced unresectable ESCC, with manageable toxicity. These findings warrant prospective validation and support biomarker-driven patient selection.

## 1 Introduction

Esophageal squamous cell carcinoma (ESCC) poses a substantial global health challenge, particularly prevalent across East Asian populations (1, 2). Definitive concurrent chemoradiotherapy (CRT) is the standard of care for patients with locally advanced unresectable disease. Despite this, long-term survival remains poor, with five-year overall survival (OS) seldom exceeding 30% and progression occurring in many patients (3–6). Over the past decade, immune checkpoint inhibitors (ICIs) have markedly improved survival outcomes in advanced or metastatic ESCC. These encouraging results have paved the way for exploring its incorporation within multimodal therapeutic regimens for locally advanced disease. Nevertheless, the combination of ICIs with definitive CRT in treating locally advanced ESCC remains unclear, and questions regarding efficacy, progression patterns, safety and prognostic determinants remain unresolved, representing a critical knowledge gap.

The development of immunotherapy in ESCC has gradually evolved from advanced disease toward earlier stages. The KEYNOTE-181, ESCORT, and ATTRACTION-3 trials established the superiority of pembrolizumab, camrelizumab, and nivolumab over chemotherapy as second-line treatment (7–9). Later phase III randomized trials, including KEYNOTE-590 and CheckMate 648, established the clinical value of combining ICIs with chemotherapy in the first-line treatment setting (10–16). More recently, immunotherapy has been extended to neoadjuvant and locally advanced settings. Trials such as ESCORT-NEO and NICE demonstrated that neoadjuvant immunochemotherapy followed by surgery could achieve high pathological response rates with manageable toxicity (17–19). These findings collectively support the shift of immunotherapy from a palliative approach toward potentially curative strategies.

Evidence from prospective clinical studies and real-world cohorts indicates that adding ICIs to definitive CRT may confer therapeutic advantages for individuals with unresectable, locally advanced ESCC. The phase II EC-CRT-001 study reported that toripalimab with CRT yielded a 62% complete response rate and a 1-year overall survival of 78.4% (20). Similarly, the GASTO 1071 study reported that neoadjuvant immunochemotherapy followed by concurrent CRT resulted in an objective response rate exceeding 90% and an 18-month progression-free survival (PFS) rate over 65% (21). Moreover, a multicenter real-world retrospective study involving 290 patients demonstrated that definitive CRT plus ICIs significantly prolonged OS compared with definitive CRT alone, with 2-year OS rates of 66.9% versus 56.5% (22). Despite these encouraging findings, existing studies have not systematically addressed how immunotherapy combined with definitive CRT influences progression patterns, the dynamic risk of disease progression, or clinical and biological prognostic factors that could guide patient selection.

Based on this rationale, the present study utilized a large single-center retrospective cohort and applied propensity score matching to minimize baseline imbalances. We systematically compared survival outcomes and progression patterns between patients receiving definitive CRT combined with immunotherapy and those treated with definitive CRT alone, and further explored independent prognostic factors and safety profiles associated with the combined modality. The study seeks to generate real-world evidence that could support optimization of therapeutic strategies and facilitate personalized treatment planning, ultimately contributing to better survival and quality of life among patients with locally advanced, unresectable ESCC.

## 2 Materials and methods

### 2.1 Data collection and time frames

We conducted a retrospective cohort study at Fujian Provincial Cancer Hospital, including patients with locally advanced unresectable ESCC treated with definitive CRT plus ICIs (CRT+ICIs group) or definitive CRT alone (CRT group) at our institution.

Patients in the CRT+ICIs group were initially treated between August 2019 and August 2024. Medical records were retrospectively collected from January to June 2025, with a final telephone follow-up in July 2025 to update survival status. The database was locked on July 31, 2025, and data processing and statistical analyses were performed from August to October 2025.

For comparison, a historical control group (CRT group) was utilized, comprising patients who were treated with definitive CRT alone between January 2012 and December 2016. Their data had been collected and archived in the hospital database as part of routine care and prior research initiatives.

### 2.2 Patient selection

Eligible patients in the CRT+ICIs group met the following conditions: (1) ESCC confirmed through histology via endoscopic biopsy; (2) clinical stage II-IVB, with M1 limited to supraclavicular lymph node metastasis (as per the 8th edition of the AJCC TNM classification); (3) relatively complete clinical data; and (4) a minimum original tumor radiation dose of ≥50 Gy. Patients with other malignancies, previous surgery for esophageal cancer, or other distant metastases at the time of initial treatment were excluded from this retrospective study.

**Figure 1:**
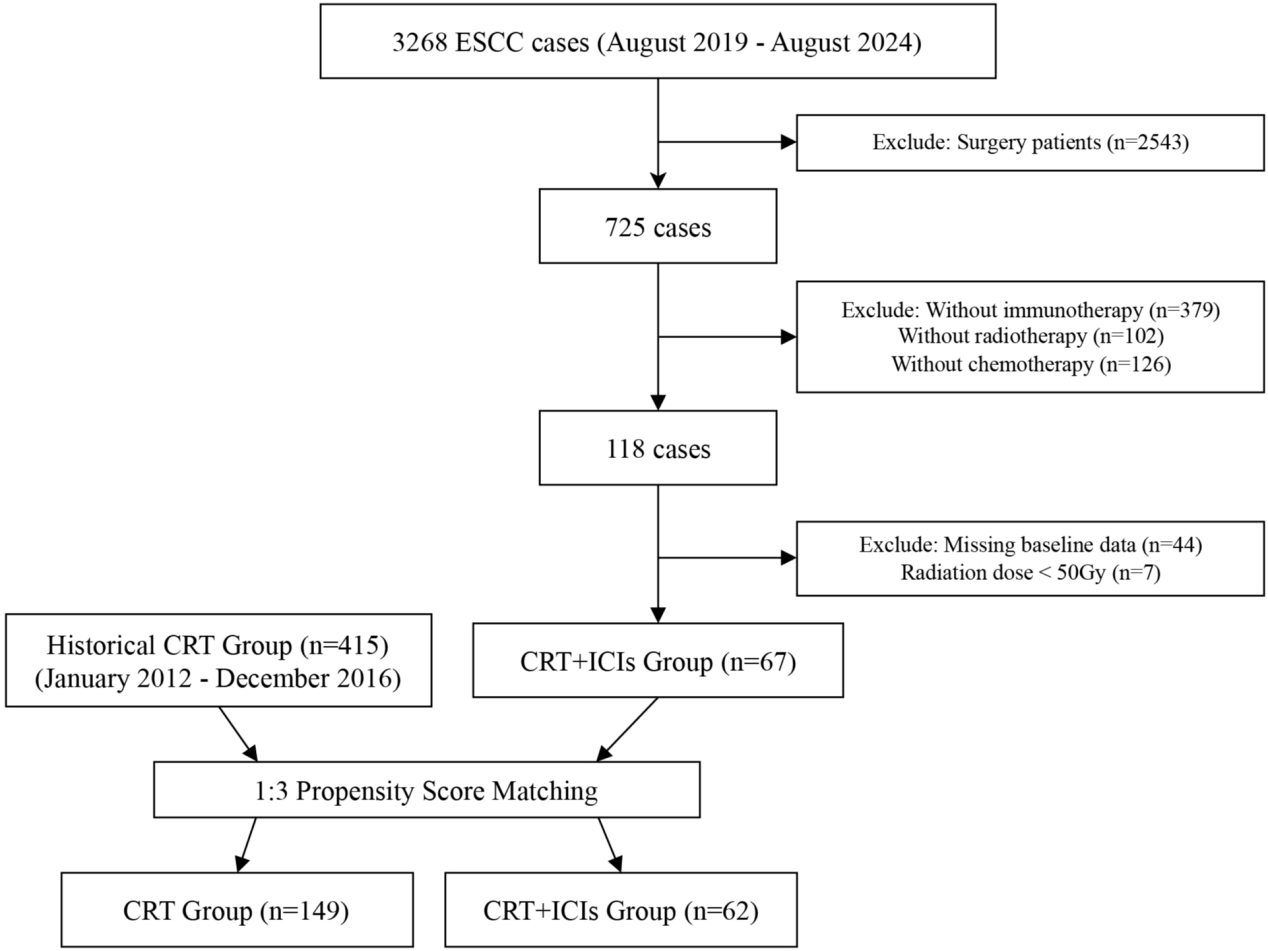
Flow diagram of patient screening and grouping.

Among 3,268 screened ESCC cases, 67 patients who underwent definitive CRT plus ICIs were identified as the CRT+ICIs group. For comparison, 415 ESCC patients treated with definitive CRT alone were included as the historical control group (CRT group) (**Figure 1**).

This study was conducted in accordance with the principles of the Declaration of Helsinki and was approved by the Ethics Committee of Fujian Cancer Hospital (K2025-254-01).

### 2.3 Treatment

Both groups underwent intensity-modulated radiotherapy (IMRT) with conventional fractionation, with a daily fraction dose ranging from 1.8 to 2.5 Gy. In the CRT group and the CRT+ICIs group, the median total radiation dose to the gross tumor volume (GTV) was 61.5 Gy (interquartile range [IQR], 60–63) and 60 Gy (IQR, 56–60), respectively. Radiation treatment planning was conducted by delineating the GTV, clinical target volume (CTV), and relevant organs at risk (OARs). The dose limitations for both target areas and OARs followed the guidelines reported in previous literature (23, 24).

In the CRT group and the CRT+ICIs group, 82.2% (341/415) and 67.2% (45/67) of patients, respectively, received doublet chemotherapy consisting of a taxane (paclitaxel or nab-paclitaxel) combined with a platinum agent (cisplatin or carboplatin). In addition, 7.5% (31/415) and 7.5% (5/67) of patients in each group received other doublet regimens. 4.3% (18/415) and 13.4% (9/67) received monotherapy. And 6.0% (25/415) and 11.9% (8/67) underwent two or more chemotherapy regimens for various reasons.

Participants in the CRT + ICIs group received a median of 4 courses of ICIs (IQR 2–6), primarily involving programmed death receptor-1 (PD-1) inhibitors with tislelizumab, pembrolizumab, toripalimab, among others.

### 2.4 Follow-up

Patients were scheduled for clinical evaluations every quarter during the initial two years following radiotherapy, every half-year from the third to fifth year, and annually beyond that. OS refers to the time span from the commencement of treatment to death from any cause, whereas PFS refers to the interval from the start of treatment to the first noted disease progression or death from any cause.

The tumor’s response was evaluated 1 to 3 months following radiotherapy using the Response Evaluation Criteria in Solid Tumors version 1.1 (RECIST v1.1). Patterns of initial disease progression were classified as local-regional progression (esophageal or regional lymph node progression) or distant progression (distant organ metastasis or nonregional lymph node involvement, including supraclavicular or para-aortic nodes, or enlargement of pre-existing supraclavicular nodes meeting RECIST criteria for progressive disease). If both local-regional and distant progression occurred simultaneously, the case was categorized as distant progression.

Retrospectively collected data also included several baseline parameters, such as maximum metastatic lymph node diameter and tumor length on X-ray (X-ray length). The Common Terminology Criteria for Adverse Events version 5.0 (CTCAE v5.0) was used to assess adverse events (AEs).

### 2.5 Hematological and biochemical parameters

As part of routine pre-treatment clinical assessments, baseline hematological and biochemical parameters of patients in the CRT + ICIs group were collected before the initiation of therapy.

Five hematologic and biochemical derived indices were analyzed: NLR (neutrophil count / lymphocyte count), LMR (lymphocyte count / monocyte count), PLR (platelet count / lymphocyte count), LAR (serum LDH (U/L) / albumin (g/L)), and the PNI calculated as albumin (g/L) + 5 × lymphocyte count (×10^9^/L).

### 2.6 Statistical analysis

Propensity score matching (PSM) was performed using a nearest-neighbor algorithm at a 1:3 ratio (CRT+ICIs: CRT), without replacement. A caliper width of 0.05 was imposed on the logit of the propensity score to restrict matches to patients with sufficiently similar baseline characteristics. Kaplan-Meier plots were created to assess OS and PFS both before and after PSM with survival differences assessed via the log-rank test. The reverse Kaplan-Meier technique was employed to compute the median follow-up period.

The Fine-Gray competing risks model was utilized for comparing local-regional and distant progression between groups (25). Proportional hazards assumptions were tested by including time-by-treatment interaction terms. Local-regional progression was analyzed with distant progression and death as competing events, whereas distant progression was analyzed with local-regional progression and death as competing events.

Optimal cutoff values for continuous variables (maximum metastatic lymph node diameter, X-ray length, NLR, LMR, PLR, LAR, and PNI) were determined by the “surv_cutpoint” package using the maximal selection rank statistics. Univariate and multivariate Cox regression analyses were utilized to pinpoint prognostic factors for OS within the CRT+ICIs group. Variables with a p-value under 0.1 from the univariate analysis, along with clinically significant factors, were incorporated into the multivariate analysis. Data analysis and figure generation were performed with R (version 4.3.0). A two-sided p value of < 0.05 was interpreted as indicating statistical significance.

## 3 Results

### 3.1 Patient characteristics

A total of 67 patients were included in the CRT+ICIs group and compared with 415 patients in the CRT group. After 1:3 PSM based on age, sex, AJCC 8th edition clinical stage, T/N classification, and tumor location, 62 patients remained in the CRT+ICIs group and 149 patients in the CRT group. Baseline characteristics were well balanced between the two groups (p > 0.05, **Table 1**). In the matched CRT+ICIs group, 79.0% were male, 77.4% presented with stage IV disease, 14.5% with stage III, and only 8.1% with stage II (**Supplementary Table 1**).

**Table 1:** Patients characteristics before and after matching based on propensity scores.

Variables	Original data set (n=482)			PSM data set (n=211)		
	CRT n=415	CRT+ICIs n=67	P value	CRT n=149	CRT+ICIs n=62	P value
Age (mean, SD)	61.01 (8.65)	65.70 (10.74)	<0.001	63.62 (7.77)	64.65 (10.37)	0.430
Gender(%)			1.000			0.748
Male	315 (75.9)	51 (76.1)		113 (75.8)	49 (79.0)	
Female	100 (24.1)	16 (23.9)		36 (24.2)	13 (21.0)	
Clinical stage			0.183			0.939
II	47 (11.3)	5 (7.5)		12 (8.1)	5 (8.1)	
III	90 (21.7)	10 (14.9)		23 (15.4)	9 (14.5)	
IVA	181 (43.6)	29 (43.3)		70 (47.0)	27 (43.5)	
IVB	97 (23.4)	23 (34.3)		44 (29.5)	21 (33.9)	
Clinical T stage			0.895			0.947
T1	1 (0.2)	0 (0.0)		0 (0.0)	0 (0.0)	
T2	29 (7.0)	3 (4.5)		10 (6.7)	3 (4.8)	
T3	125 (30.1)	19 (28.4)		40 (26.8)	17 (27.4)	
T4a	34 (8.2)	5 (7.5)		10 (6.7)	5 (8.1)	
T4b	226 (54.5)	40 (59.7)		89 (59.7)	37 (59.7)	
Clinical N stage			0.020			0.998
N0	110 (26.5)	9 (13.4)		22 (14.8)	9 (14.5)	
N1	163 (39.3)	24 (35.8)		54 (36.2)	23 (37.1)	
N2	120 (28.9)	31 (46.3)		65 (43.6)	27 (43.5)	
N3	22 (5.3)	3 (4.5)		8 (5.4)	3 (4.8)	
Tumor location			0.058			0.916
Cervical	47 (11.3)	13 (19.4)		26 (17.4)	13 (21.0)	
Upper	130 (31.3)	19 (28.4)		45 (30.2)	17 (27.4)	
Middle	198 (47.7)	24 (35.8)		56 (37.6)	22 (35.5)	
Lower	40 (9.6)	11 (16.4)		22 (14.8)	10 (16.1)	

### 3.2 Survival outcomes

**Figure 2:**
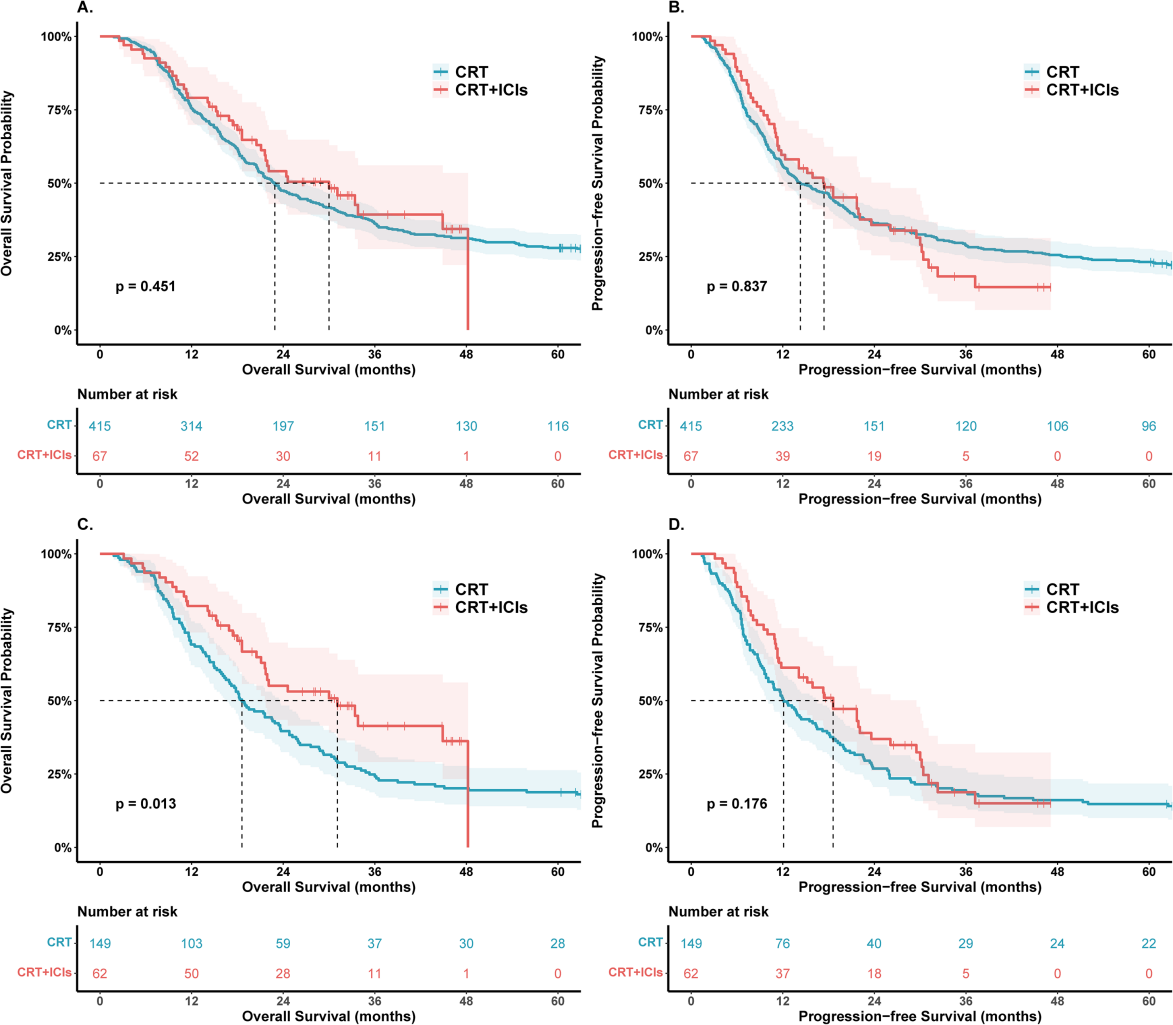
**(A–D)** Kaplan-Meier curves for OS **(A, C)** and PFS **(B, D)** of patients before **(A, B)** and after **(B, C)** propensity score matching. Shaded areas represent 95% confidence intervals estimated from the log hazard. The numbers of patients at risk at different time points and log-rank p values are shown on each plot.

The median follow-up time was 33.0 months (95% CI: 29.3–36.7) in the CRT+ICIs group and 90 months (95% CI: 85.0–96.0) in the CRT group. Kaplan-Meier analysis found no significant OS (p = 0.451) or PFS (p = 0.837) difference before PSM (**Figures 2A, B**). After PSM, the median OS of the CRT+ICIs group rose to 31.1 months (95% CI: 21.6–NA), which was significantly longer than the CRT group’s 18.6 months (95% CI: 16.5–23.6) (p = 0.013, **Figure 2C**). Median PFS was 18.6 months (95% CI: 14.1–29.5) in the CRT+ICIs group versus 12.1 months (95% CI: 10.1–16.4) in the CRT group, with a nonsignificant trend toward improvement (p = 0.176, **Figure 2D**).

After PSM, the 1-, 2-, and 3-year OS rates were 82.2% (95% CI: 73.2–92.3), 55.1% (95% CI: 43.5–69.8), and 41.4% (95% CI: 29.1–58.9) in the CRT+ICIs group, compared with 69.1% (95% CI: 62.1-77.0), 39.6% (95% CI: 32.5-48.3), and 24.2%(95% CI: 18.2-32.1) in the CRT group, respectively. The corresponding 1-, 2-, and 3-year PFS rates were 61.2% (95% CI: 50.2-74.6), 36.9% (95% CI: 26.1-52.1), and 18.8% (95% CI: 10.0-35.2) in the CRT+ICIs group versus 50.3% (95% CI: 42.9-59.0), 26.8% (95% CI: 20.6-35.0), 18.8% (95% CI: 13.5-26.2) in the CRT group (**Supplementary Table 2**).

### 3.3 Patterns of initial disease progression

Disease progression occurred in 76 of 149 patients (51.0%) in the CRT group and 26 of 62 patients (41.9%) in the CRT+ICIs group. In the CRT group, local-regional progression accounted for 36.8% (28/76) and distant metastasis for 63.2% (48/76). In the CRT+ICIs group, 46.2% (12/26) experienced local-regional progression and 53.8% (14/26) had distant progression (**Supplementary Table 3**).

**Figure 3:**
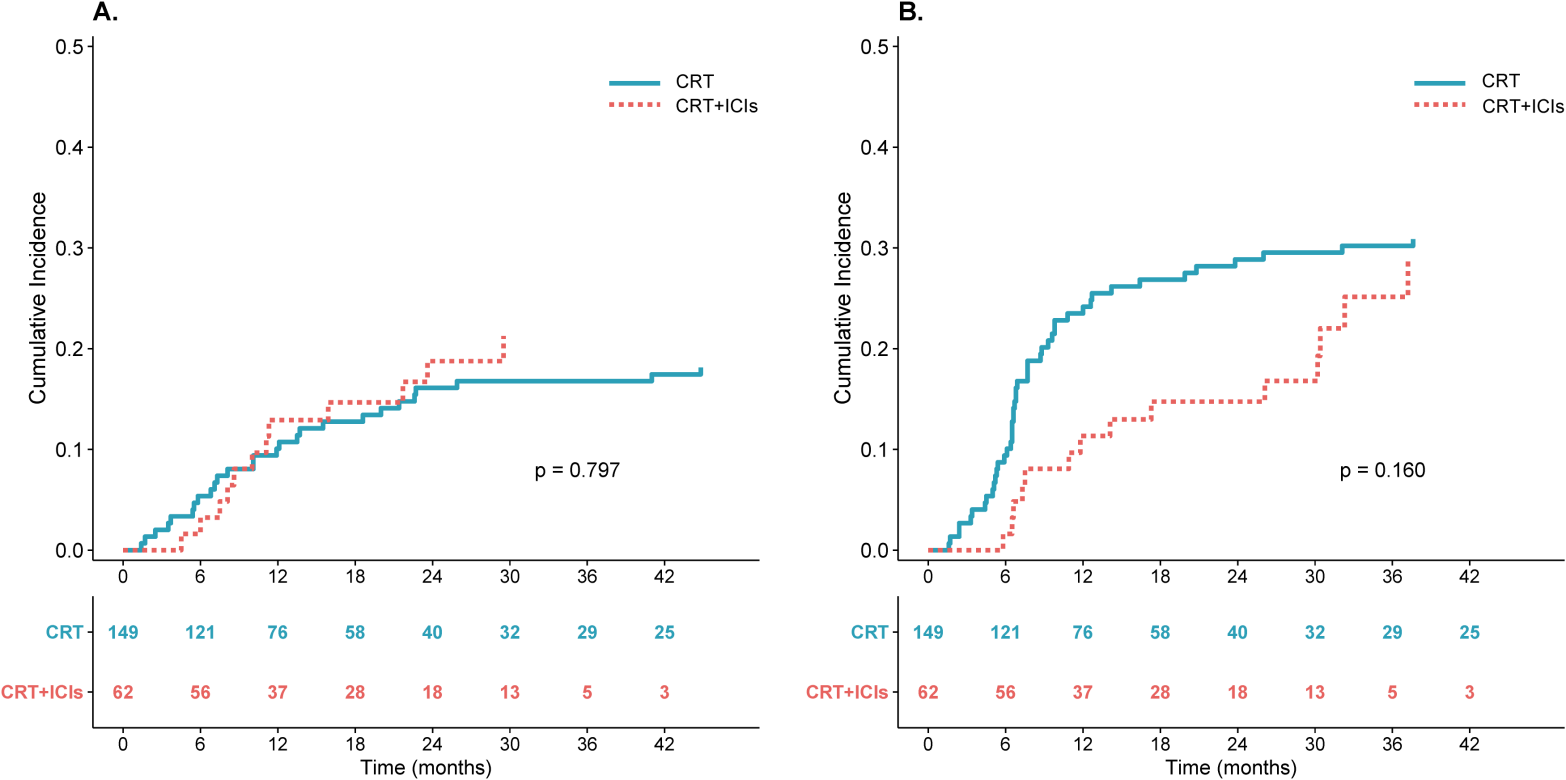
Cumulative incidence curves of local-regional progression **(A)** and distant progression **(B)** after PSM between the CRT and CRT+ICIs groups.

Time-dependent effect analysis found a significant interaction term for local-regional progression (p = 0.034), suggesting violation of the proportional hazards assumption. In the early treatment phase, the CRT+ICIs group exhibited a higher risk of local-regional progression compared with the CRT group (baseline HR = 3.130, 95% CI: 1.052–9.312, p = 0.040). However, the risk declined over time (interaction coefficient β = –0.0654, p = 0.034), with both groups showing equal risk at approximately 17.5 months post-treatment. Beyond this timepoint, CRT+ICIs was associated with a lower risk of local-regional progression. Cumulative incidence curves are shown in **Figure 3A**, and time-varying hazard ratios are depicted in **Supplementary Figure 1**.

For distant progression, the interaction term was not significant (p = 0.510), supporting the proportional hazards assumption. Fine-Gray model analysis found a 34% risk reduction trend in the CRT+ICIs group (subdistribution HR = 0.66, 95% CI: 0.37–1.18), although this was not statistically significant (p = 0.160) (**Figure 3B**).

### 3.4 Prognostic factors for survival

Univariate and multivariate Cox regression analyses were performed in the matched CRT+ICIs group to find prognostic factors for OS. Continuous variables, including maximum metastatic lymph node diameter, X-ray length, NLR, LMR, PLR, LAR, and PNI were dichotomized based on optimal cutoff values (**Table 2**).

**Table 2:** Cox regression results for overall survival in the CRT + ICIs group after propensity score matching.

Characteristic	Univariate analysis		Multivariate analysis	
	Hazard ratio (95% CI)	P value	Hazard ratio (95% CI)	P value
Age				
< 65	Reference			
≥ 65	1.21 (0.57-2.55)	0.623		
Gender				
Female	Reference			
Male	1.06 (0.46-2.45)	0.899		
Tumor location				
Cervical	Reference			
Upper	0.94 (0.35-2.53)	0.900		
Middle	0.87 (0.34-2.24)	0.767		
Lower	0.92 (0.29-2.92)	0.894		
X-ray length, cm				
< 3.8	Reference			
≥ 3.8	1.42 (0.69-2.92)	0.339		
Max met-LN diameter				
< 2.8	Reference			
≥ 2.8	2.25 (0.97-5.22)	0.059	1.33 (0.39-4.49)	0.649
GTV dose				
< 60	Reference			
≥ 60	1.10 (0.54-2.22)	0.796		
Induction ICIs				
No	Reference			
Yes	1.18 (0.53-2.63)	0.687		
Concurrent ICIs				
No	Reference			
Yes	1.17 (0.54-2.54)	0.690		
Adjuvant ICIs				
No	Reference	0.516		
Yes	0.79 (0.40-1.59)			
Total ICIs cycle				
< 10	Reference			
≥ 10	0.36 (0.11-1.19)	0.094	0.40 (0.10-1.68)	0.213
Clinical T stage				
T2-3	Reference			
T4a-4b	0.59 (0.29-1.23)	0.158	0.48 (0.21-1.10)	0.085
Clinical N stage				
N0-1	Reference			
N2-3	1.71 (0.84-3.45)	0.137	1.07 (0.47-2.42)	0.877
Clinical M stage				
M0	Reference			
M1	2.13 (1.06-4.27)	0.033	2.97 (1.24-7.09)	0.014
NLR				
< 3.1	Reference			
≥ 3.1	0.54 (0.26-1.13)	0.101		
LMR				
< 3.1	Reference			
≥ 3.1	0.37 (0.16-0.84)	0.018	0.90 (0.28-2.93)	0.866
PLR				
< 205.4	Reference			
≥ 205.4	3.09 (1.44-6.64)	0.004	1.40 (0.45-4.36)	0.561
LAR				
< 4.1	Reference			
≥ 4.1	3.16 (1.21-8.24)	0.019	2.75 (1.03-7.33)	0.043
PNI				
< 47.9	Reference			
≥ 47.9	0.40 (0.19-0.83)	0.014	0.38 (0.16-0.94)	0.036
Efficacy after RT				
CR/PR	Reference			
SD/PD	0.56 (0.27-1.16)	0.117		

Max met-LN diameter, maximum metastatic lymph node diameter; GTV, gross tumor volume; ICIs, immune checkpoint inhibitors; NLR, neutrophil-to-lymphocyte ratio; LMR, lymphocyte-to-monocyte ratio; PLR, platelet-to-lymphocyte ratio; LAR, lactate dehydrogenase to albumin ratio; PNI, prognostic nutritional index, serum albumin (g/L) + 5 × total lymphocytes (×10^9^/L); RT, radiation treatment; CR, complete response; PR, partial response; SD, stable disease; PD, progressive disease.

**Figure 4:**
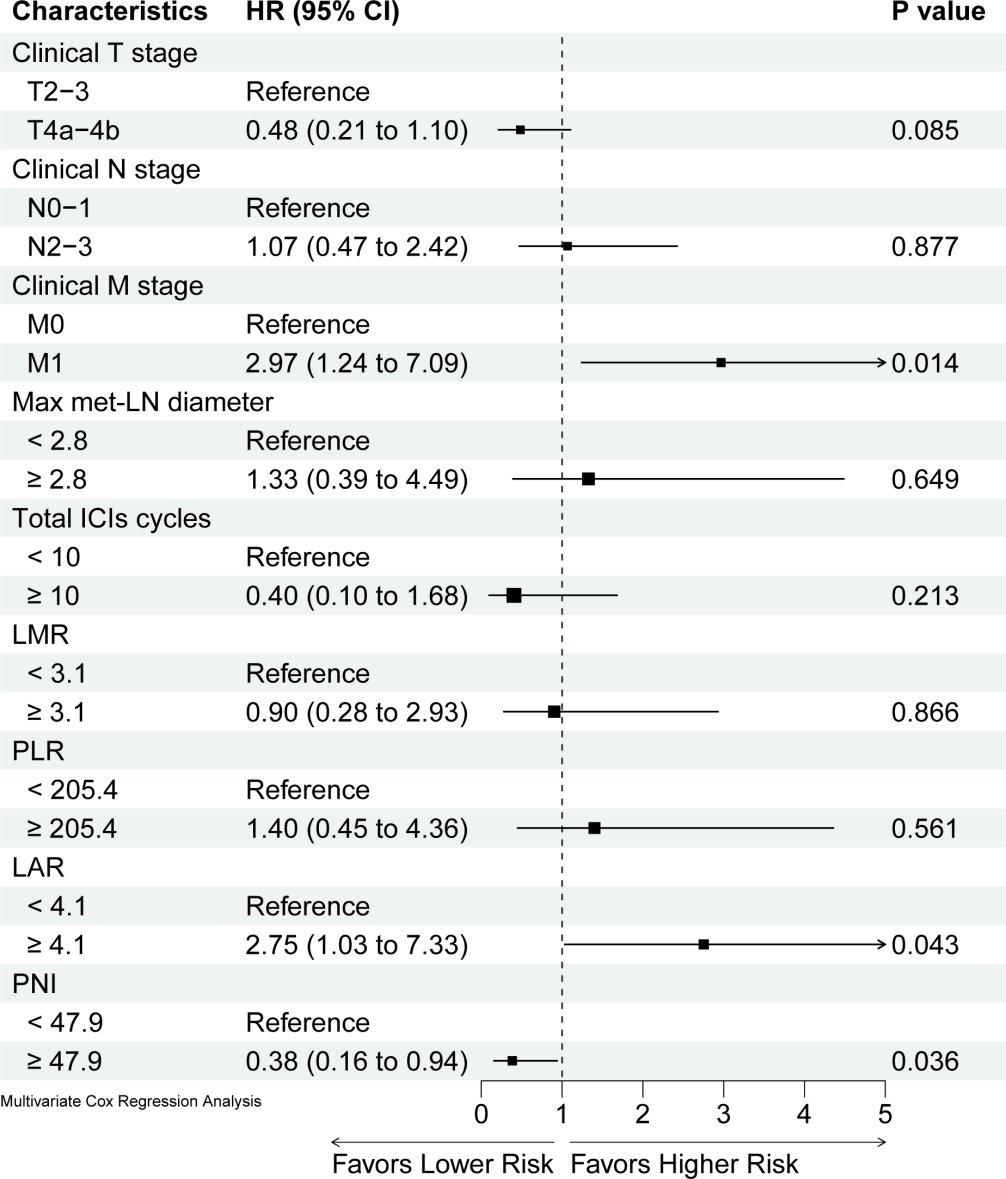
Forest plot of multivariate Cox regression analysis of prognostic factors in the CRT+ICIs group.

Univariate analysis identified M stage, LMR, PLR, LAR, and PNI as significant prognostic factors (p < 0.05). For the multivariate analysis, variables with p-values less than 0.1 from the univariate analysis and clinically relevant parameters (T and N stage) were considered. Multivariate analysis demonstrated that M stage, LAR, and PNI were independent prognostic factors for OS. Specifically, PNI ≥ 47.9 was associated with a protective effect, whereas M1 disease and LAR ≥ 4.1 were adverse prognostic indicators. A forest plot of multivariate results is presented in **Figure 4**.

### 3.5 Adverse events

Among the 62 patients treated with CRT+ICIs, grade ≥ 3 AEs occurred in 38.7% (24/62), primarily hematologic toxicities and radiation-related events (**Table 3**). Hematologic toxicities included leukopenia (10/62, 16.1%), neutropenia (11/62, 17.8%), and anemia (2/62, 3.2%). Grade 3 radiation esophagitis occurred in 11.3% (7/62).

**Table 3:** Adverse events (n=62).

Adverse events	NCI-CTCAE grade			
	Grade 1	Grade 2	Grade 3	Grade 4
Bilirubin increased	28 (45.2)	2 (3.2)	0 (0)	0 (0)
ALT increased	14 (22.6)	5 (8.1)	2 (3.2)	0 (0)
AST increased	19 (30.6)	2 (3.2)	2 (3.2)	0 (0)
GGT increased	15 (24.2)	5 (8.1)	0 (0)	0 (0)
ALP increased	20 (32.3)	1 (1.6)	0 (0)	0 (0)
Creatinine increased	3 (4.8)	2 (3.2)	0 (0)	0 (0)
Leukopenia	10 (16.1)	25 (40.3)	9 (14.5)	1 (1.6)
Neutropenia	12 (19.4)	10 (16.1)	7 (11.3)	4 (6.5)
Anemia	27 (43.5)	13 (21.0)	1 (1.6)	1 (1.6)
Thrombocytopenia	16 (25.8)	5 (8.1)	2 (3.2)	0 (0)
Radiation pneumonitis	11 (17.7)	6 (9.7)	1 (1.6)	0 (0)
Radiation esophagitis	5 (8.1)	24 (38.7)	7 (11.3)	0 (0)
Esophageal fistula	0 (0)	1 (1.6)	0 (0)	0 (0)
Esophageal stricture	4 (6.5)	2 (3.2)	1 (1.6)	0 (0)
Pleural effusion	13 (21.0)	0 (0)	0 (0)	0 (0)
Pericardial effusion	1 (1.6)	12 (19.4)	0 (0)	0 (0)
Myocardial injury	0 (0)	1 (1.6)	0 (0)	0 (0)
Arrhythmia	1 (1.6)	1 (1.6)	0 (0)	0 (0)
Heart failure	0 (0)	1 (1.6)	1 (1.6)	0 (0)
Immune-related adverse events				
Hypertriglyceridemia	27 (43.5)	3 (4.8)	0 (0)	0 (0)
Hyperthyroidism*	11 (17.7)	0 (0)	0 (0)	0 (0)
Hypothyroidism*	6 (9.7)	5 (8.1)	0 (0)	0 (0)
Rash	0 (0)	0 (0)	1 (1.6)	0 (0)
Immune-related pneumonitis	0 (0)	1 (1.6)	0 (0)	0 (0)

Values are presented as number (%).

*including subclinical state.

NCI-CTCAE, National Cancer Institute Common Terminology Criteria for Adverse Events version 5.0; ALT, alanine aminotransferase; AST, aspartate aminotransferase; GGT, gamma-glutamyltransferase; ALP, alkaline phosphatase.

Notably, grade 4 events were confined to hematologic toxicities, including neutropenia (4/62, 6.5%), leukopenia (1/62, 1.6%), and anemia (1/62, 1.6%). Immune-related adverse events (irAEs) were generally mild, with only one case of grade 3 rash (1.6%) and no grade ≥ 3 endocrine or pulmonary toxicities. 2 patients (3.2%) discontinued immunotherapy due to irAEs, one with grade 3 rash and the other with grade 2 immune-related pneumonitis.

## 4 Discussion

This retrospective cohort study evaluated the clinical efficacy, progression patterns of definitive CRT+ICIs versus definitive CRT alone in patients with locally advanced unresectable ESCC. Using PSM to balance baseline characteristics, we demonstrated that the addition of ICIs to CRT significantly improved OS and showed a trend toward prolonged PFS. Furthermore, The Fine-Gray model indicated that combination therapy was associated with a nonsignificant trend toward a reduced risk of distant progression, and multivariate Cox regression identified M stage, LAR, and PNI as independent prognostic factors of OS. The combination therapy exhibited an acceptable safety profile, with irAEs generally mild and manageable.

Our study found that the CRT+ICIs group reached a median OS of 31.1 months compared with 18.6 months in the CRT group after PSM, with 1-, 2-, and 3-year OS rates of 82.2%, 55.1%, and 41.4%, respectively. These findings are consistent with and extend prior evidence from phase II and real-world studies. In the EC-CRT-001 trial, the combination of toripalimab with definitive CRT led to a 1-year OS rate of 78.4% (20), while Yang et al. recently reported 1- and 2-year OS rates of 86.7% and 66.9% for definitive CRT plus ICIs in a multicenter real-world cohort (22). Differences in results across different studies may be attributed to variations in the proportion of patients with stage IV disease included. The magnitude of survival benefit observed in our study reinforces the growing body of evidence supporting the synergistic potential of ICIs with CRT in unresectable ESCC. Mechanistically, tumor cells exposed to chemoradiotherapy (CRT) may undergo immunogenic cell death, which facilitates the exposure of tumor-associated antigens and subsequently stimulates antitumor immune responses. In some patients, this process may trigger an “abscopal effect”, in which localized radiation elicits systemic immune responses against distant tumor sites (26–28). Together, these mechanisms provide a biological rationale for the synergistic potential of combining ICIs with CRT, potentially reshaping the survival landscape for patients with locally advanced unresectable ESCC.

The progression risk analysis in our study provides novel insight into initial progression patterns under combined modality treatment. Initially, the CRT+ICIs group showed a transiently higher risk of local-regional progression compared with CRT alone. However, this risk declined over time and became lower after approximately 17.5 months. Secondly, compared with CRT alone, the CRT + ICIs group showed a 34% relative decrease in distant progression risk, though the variation did not achieve statistical significance. And The combination group had a less 12-month cumulative distant progression rate compared to the CRT group (11.3% vs. 24.2%), which is consistent with the 1-year distant metastasis-free survival rate of 84.8%–85.1% observed in the GASTO 1071 trial of neoadjuvant immunochemotherapy followed by concurrent chemoradiotherapy (21).

Over the longer term, however, the difference of distant progression between the two groups tended to narrow, possibly due to reduced treatment cycles or discontinuation of immunotherapy, thereby dampening the maintenance effect of ICIs. Consistently, organ-specific patterns of first distant progression were descriptively examined (**Supplementary Table 3**): lung (11.5% vs. 13.2%), liver (3.8% vs. 3.9%), brain (3.8% vs. 2.6%), and multi-organ metastases (7.7% vs. 9.2%) were similar between CRT+ICIs and CRT group, whereas bone metastasis appeared numerically higher (7.7% vs. 2.6%) and other sites were lower (3.8% vs. 13.2%). Although exploratory and potentially influenced by small event numbers and surveillance differences, these findings suggest that ICIs may primarily contribute to systemic control by suppressing micrometastatic dissemination, while certain metastatic niches (e.g., bone) may remain relatively permissive (29). Meanwhile, the delayed yet durable local-regional control may reflect progressive immune reactivation and tumor microenvironment remodeling following CRT-induced antigen exposure (30). Such progression model provides a refined understanding beyond static risk estimates, underscoring the need for longitudinal assessment in future clinical studies.

In the CRT+ICIs group, results from the multivariate Cox regression showed that M stage, PNI, and LAR, were closely associated with OS (P < 0.05). Among these factors, a PNI ≥ 47.9 had a protective impact, while an LAR ≥ 4.1 and M1 stage indicated worse prognosis. The formula for PNI is albumin (g/L) + 5×peripheral lymphocyte count (10^9^/L). A low PNI indicates impaired immune competence and nutritional deficiency, both of which may compromise the antitumor immune response. And it was initially used to evaluate the immunonutritional condition and surgical risk in gastrointestinal operations. More recently, it has proven to be closely related to prognosis in multiple solid malignancies (31–34). The LAR simultaneously indicates tumor metabolic activity, overall nutritional status, and inflammatory response. Previous research in operable esophageal cancer found that a high preoperative LAR consistently correlated with poorer survival outcomes. Studies by Feng et al. (cutoff: 5.5) and Shiratori et al. (cutoff: 6.2) confirmed that a high LAR remained an independent prognostic factor for patients undergoing radical resection, irrespective of neoadjuvant treatment (35, 36). These readily available and practical hematologic indicators provide clinicians with useful references for further stratified patient management.

Adverse events in this study was manageable and comparable to previously published findings. In the CRT+ICIs group, 38.7% of patients experienced grade 3 or higher adverse events, primarily hematologic and radiation-related toxicities. These findings are consistent with prior reports in the literature, in which myelosuppression and radiation-induced esophagitis were commonly observed during CRT (3–5). Importantly, severe irAEs were infrequent (3.2%), with only one case of grade 3 rash and no grade ≥3 endocrine or pulmonary toxicity observed. These data align with the EC-CRT-001 study, where 69% of patients experienced irAEs, mostly grade 1–2 events such as hypothyroidism and rash (20), and support the acceptable tolerability of this regimen in the definitive setting. The absence of unexpected safety signals indicates that ICIs can be safely integrated into CRT protocols for unresectable ESCC, provided that close monitoring and multidisciplinary management are implemented.

This study provides several novel insights to the current understanding of multimodal immunotherapy for ESCC. First, it provides real-world, PSM-adjusted evidence that adding ICIs to definitive CRT confers a meaningful survival benefit in patients with locally advanced unresectable ESCC. Second, by integrating time-dependent competing risk analysis and Fine-Gray subdistribution hazards model, this study elucidated the dynamic evolution of local-regional progression risk under immune modulation, and suggests a trend toward reduced distant progression risk—an aspect rarely explored in previous literature. Third, it identifies clinically practical prognostic factors (LAR, PNI, and M stage) that can aid in patient selection and treatment optimization. Collectively, these findings validate the efficacy and tolerability of CRT plus ICIs in routine clinical practice and highlight the potential of integrating simple prognostic indices into risk-stratified treatment algorithms.

Several limitations should be acknowledged. First, being a retrospective analysis conducted at a single center, the study may suffer from selection bias and constrained generalizability, despite adjustment through PSM. Second, heterogeneity in ICIs types and chemotherapy regimens might have influenced treatment outcomes. Third, the lack of biomarker data (such as programmed death-ligand 1 status, mutational load, and immune-related transcriptomic profiles) restricted further exploration of immunologic mechanisms and predictive markers. Additionally, the relatively short follow-up for some patients may underestimate long-term survival and late-onset irAEs. Future prospective, multicenter studies incorporating molecular profiling and standardized immunotherapy protocols are warranted to validate these findings. Furthermore, integrating radiomic or immune-nutritional indicators could refine prognostic models and guide adaptive treatment strategies. Despite these inherent limitations, our investigation substantiates the practicality and efficacy of definitive CRT combined with ICIs in locally advanced unresectable ESCC.

## 5 Conclusions

In conclusion, compared with CRT alone, definitive CRT combined with ICIs confers a marked improvement in OS for patients with locally advanced unresectable ESCC. It also shows a clinical trend in reducing the risk of early distant progression, without significantly increasing severe toxicities. Prognostic nutritional and biochemical indices such as PNI and LAR hold predictive value for treatment response. As prospective large-cohort studies progress, definitive CRT combined with ICIs is poised to become an important standard treatment for this population. Further evidence is needed to determine the optimal radiation dose and to refine biomarker selection for a more precise, individualized comprehensive treatment strategy.

## Data Availability

The raw data supporting the conclusions of this article will be made available by the authors, without undue reservation.

## Supplementary material

Supplementary Table 1 Characteristics of patients (n = 62).

Supplementary Table 2 Survival rates of patients stratified by treatment methods.

Supplementary Table 3 Initial progression classification.

Supplementary Figure 1 Time-dependent curves for local-regional progression between the CRT+ICIs and CRT groups after PSM. The black line represents the estimated hazard ratio (HR) over time according to HR(t) = exp(1.1411 − 0.0654 × t).
