# Supplementary material for: Definitive chemoradiotherapy plus immune checkpoint inhibitors for locally advanced unresectable esophageal squamous cell carcinoma: survival and progression patterns in a propensity‐matched cohort
Source: Front Immunol. 2026 May 28;17:1778539. doi: 10.3389/fimmu.2026.1778539 (PMC13253816; doi:10.3389/fimmu.2026.1778539)
Supplement: Supplementary Table 1 — Characteristics of patients (n = 62). [file DataSheet1.pdf]

Supplementary Material

1 Supplementary Figures and Tables

1.1 Supplementary Tables

Supplementary Table 1. Characteristics of patients (n = 62).

Characteristic	Value
Age (median (range)), years	67 (26-84)
Gender	
Male	13 (21.0%)
Female	49 (79.0%)
Tumor location	
Cervical	13 (21.0%)
Upper	17 (27.4%)
Middle	22 (35.5%)
Lower	10 (16.1%)
Clinical T stage	
T2-3	20 (32.3%)
T4a-4b	42 (67.7%)
Clinical N stage	
N0-1	32 (51.6%)
N2-3	30 (48.4%)
Clinical M stage	

M0	41 (66.1%)
M1	21 (33.9%)
Clinical stage	
II	5 (8.1%)
III	9 (14.5%)
IVA	27 (43.5%)
IVB	21 (33.9%)
Max met-LN diam (median (IQR)), cm	1.45 (0.9-2.3)
X-ray length (median (IQR)), cm	4.5 (3.5-5.9)
GTV dose (median (IQR)), Gy	60 (56-60)
Induction immunotherapy	
No	16 (25.8%)
Yes	46 (74.2%)
Concurrent immunotherapy	
No	45 (72.6%)
Yes	17 (27.4%)
Adjuvant immunotherapy	
No	31 (50.0%)
Yes	31 (50.0%)
Total ICIs cycles (median (IQR))	4 (2-6)
Total chemotherapy cycles (median (IQR))	4 (3-5)

NLR (median (IQR))	2.3 (1.9-3.3)
LMR (median (IQR))	4.3 (3.6-5.5)
PLR (median (IQR))	152.6 (117.0-184.6)
LAR (median (IQR))	4.6 (4.1-5.5)
PNI (median (IQR))	48.4 (44.3-51.1)

Max met-LN diam = maximum metastatic lymph node diameter; IQR = interquartile range; GTV = gross tumor volume; Gy = gray; ICIs = immune checkpoint inhibitors; NLR = neutrophil-to-lymphocyte ratio; LMR = lymphocyte-to-monocyte ratio; PLR = platelet-to-lymphocyte ratio; LAR = lactate dehydrogenase to albumin ratio; PNI = prognostic nutritional index.

Supplementary Table 2. Survival rates of patients stratified by treatment methods.

Variables	Original data set(n=482)			PSM data set(n=211)		
	CRT n=415	CRT+ICIs n=67	P value	CRT n=149	CRT+ICIs n=62	P value
OS rate (95%CI)			0.451			0.013
6 months	95.9% (94.0-97.8%)	92.5% (86.5-99.0%)		94.0% (90.2-97.9%)	93.5% (87.6-99.9%)	
1 year	75.4% (71.4-79.7%)	79.1% (69.9-89.5%)		69.1% (62.1-77.0%)	82.2% (73.2-92.3%)	
2 years	47.5% (42.9-52.4%)	54.1% (42.9-68.1%)		39.6% (32.5-48.3%)	55.1% (43.5-69.8%)	
3 years	36.1% (31.8-41.1%)	39.3% (27.6-56.0%)		24.2% (18.2-32.1%)	41.4% (29.1-58.9%)	

PFS rate (95%CI)			0.837			0.176
6 months	81.9%	88.1%		81.2%	88.7%	
	(78.3-85.7%)	(80.6-96.2%)		(75.2-87.7%)	(81.2-96.9%)	
1 year	55.7%	59.6%		50.3%	61.2%	
	(51.1-60.7%)	(49.0-72.6%)		(42.9-59.0%)	(50.2-74.6%)	
2 years	36.4%	35.7%		26.8%	36.9%	
	(32.0-41.3%)	(25.5-50.1%)		(20.6-35.0%)	(26.1-52.1%)	
3 years	28.7%	18.2%		18.8%	18.8%	
	(24.6-33.4%)	(9.8-34.0%)		(13.5-26.2%)	(10.0-35.2%)	

Supplementary Table 3. Initial progression classification.

Patterns of progression	CRT (n = 76)	CRT+ICIs (n = 26)
Local-regional	28 (36.8%)	12 (46.2%)
Distant	48 (63.2%)	14 (53.8%)
Supraclavicular/Retroperitoneal LN	14 (18.4%)	4 (15.4%)
Lung	10 (13.2%)	3 (11.5%)
Liver	3 (3.9%)	1 (3.8%)
Bone	2 (2.6%)	2 (7.7%)
Brain	2 (2.6%)	1 (3.8%)
Other	10 (13.2%)	1 (3.8%)
Multi-organ metastases	7 (9.2%)	2 (7.7%)

LN = Lymph nodes.

1.2 Supplementary Figure

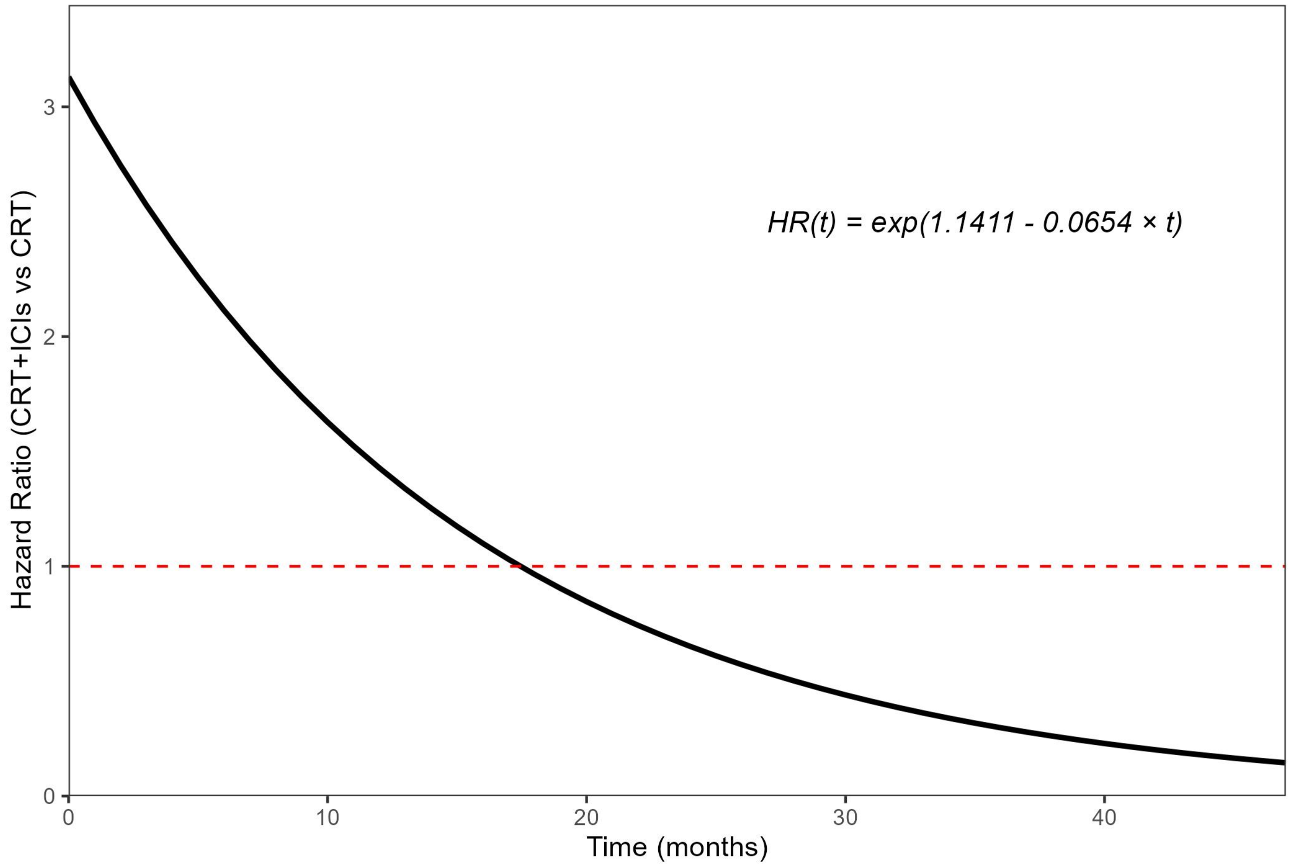

Supplementary Figure 1. Time- dependent curves for local-regional progression between the CRT+ICIs and CRT groups after PSM. The black line represents the estimated hazard ratio (HR) over time according to $HR(t) = exp(1.1411 - 0.0654 \times t)$.
